# A digital way to assess the stain parameters that lead to soft tissue blanching when delivering an implant-supported crown

**DOI:** 10.1186/s40729-025-00598-7

**Published:** 2025-02-10

**Authors:** Serge Szmukler-Moncler, David Morales Schwarz, Jorge Manuel Perez, Florian Beuer

**Affiliations:** 1 Department of Prosthodontics, Charité University of Medicine, Charité Center 03, Assmannshauser Str. 4-6, 14197 Berlin, Germany; 2 Berlin Implantology Research Group, Eichhornstrasse 02, 10785 Berlin, Germany; 3 Dental Clinic M&M, C. Estadio, 1, Valladolid, 47006 Spain

## Abstract

**Background:**

Dental implant systems provide standard cylindrical healing abutments of various diameters; however, they do not match the larger shape of the complex emergence profile of the prosthetic crowns. Adaptation of the soft tissues from a circular emergence profile to the one that suits the prosthetic crown involves a simultaneous squeezing and stretching of the gingiva. Often, this translates into local blanching of the gingiva and the prosthodontist must assess that blanching is transient. There is no literature about how much strain exerted by the prosthetic crown is leading or not to gingiva blanching. Aim of this paper is to present a digital workflow that allows measuring, upon prosthesis delivery, how much the strained gingiva is displaced under the crown and leads or not to blanching of the peri-implant soft tissues.

**Method and Results:**

The digital workflow involves 3 intra-oral scans (IOS), IOS#1 at completion of the soft tissue healing, IOS#2 at prosthesis delivery, IOS#3 after soft tissue conditioning, and the STL files of the healing cap, the abutment, the implant and the prosthetic crown. The above are superposed and merged following a dedicated protocol that provides access to the distance the delivered crown deforms the strained gingiva. The present case study displayed distinct blanching intensities. Severe blanching was present when the strains applied to the gingiva caused a displacement of 1.3 mm and above; a displacement of 0.9 mm led to moderate blanching. No blanching was observed up to a displacement of 0.6 mm.

**Conclusion:**

A digital protocol, involving the superposition and merging of IOSs taken along a defined timeline and STLs of the implant hardware, allowed measuring the displacement distances a prosthetic crown wields upon delivery on the gingiva beneath the prosthesis. Various intensities of gingiva blanching could be related to distinct displacement distances of the healed gingiva that were triggered by attaching a prosthetic crown to the implant neck.

## Background

Dental implant therapy can be handled according to a 1- or 2-stage surgery protocol. In both cases, the soft tissues are left to heal in a transgingival mode either immediately after implantation or after the second surgery following osseointegration. A healing abutment is then placed on top of the implant and protrudes in the oral cavity for a period of 6 to 12 weeks when a 1-stage is chosen [1] or 6–8 weeks after the second surgery [2]. During this time, the gingiva heals and matures against the transgingival abutment. Dental implant systems are offering conventional round healing abutments of various heights and diameters; they are far from matching the wider and complex shape of the emergence profile of prosthetic crowns [3, 4].

The orofacial and mesio-distal dimensions of the crowns of the molars and maxillary central incisors sites to rehabilitate are much larger than the rounded healing abutment [5, 6]. Under these circumstances, when the temporary or definitive crown is delivered, the fragile peri-implant mucosa undergoes heavy constraints in all directions. Local compression and stretching of the soft tissues may lead to blanching of the gingiva [5–11]; this step may be painful for the patient and often requires a local anesthesia. Blanching is the result of local ischemia; it should be resolved after 10 to 20 min [5, 8, 12–14], otherwise it may lead to oedema, inflammation, recession [12] and up to soft tissue necrosis [5, 12].

The application of uncontrolled stresses may increase the esthetic and functional risks for inadequate mucosa architecture [6, 11]; however, how much pressure leads to moderate, severe or hazardous blanching is a question that has never been addressed in the literature. The reason is that there was not, until now, a way to measure it clinically.

The aim of the present paper is to present a straightforward digital protocol that enables measuring the distance the crown is straining the delicate peri-implant mucosa and leads to moderate or severe gingival blanching or no blanching at all. A clinical case is presented to illustrate the digital methodology and to provide first results.

## Description of the digital protocol

A clinical case was chosen to present the step-by-step procedures that enables measuring the pressure exerted on the gingiva by the implant-supported crown.

### Presentation of the case

A 64-year-old male patient attended to rehabilitate his edentulous first left mandibular molar site (#36) that displayed a thick biotype gingiva. After clinical and 3D radiological examination implant therapy involving a 2-stage surgery protocol was proposed; it consisted into placing a Top DM implant of 4.0 × 10 mm (Bioner, San Just Desvern, Spain) in a 1.5 mm subcrestal position. The patient signed an informed consent to allow his data serve a publication purpose on the condition of anonymity.

The implant is designed with an internally indexed hexagonal conical connection [15] and presents a regular honeycomb like macro- and microsurface texture obtained by etching only, without sandblasting [16]. After achieving a primary stability superior to 35 Ncm, the implant was left to heal for 3 months in a submerged fashion. Three months later, the implant was uncovered and standard clinical and radiologic examinations assessed its osseointegration. A transepithelial abutment of 3 mm in height designed with a hexagonal head was screwed into the implant neck with a 25 Ncm torque. This abutment served simultaneously as a transgingival healing abutment while receiving a healing cap, and as a prosthetic abutment while receiving a screw-retained crown over its hexagonal head.

**Fig. 1:**
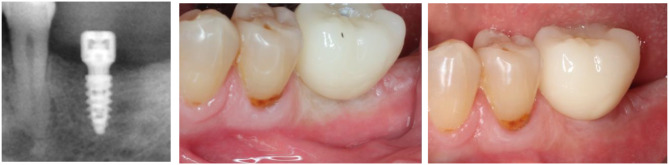
Presentation of the case. **a**) Radiographic control of the implant by the end of the soft tissue healing period. Note the bone grown over the shoulder of the Top DM implant, in contact with the concave shape of the transepithelial abutment. **b**) Blanching of the gingiva upon delivery of the implant-supported crown. Note the various blanching intensities. **c**) Vanished blanching that led the patient to be safely discharged

**Fig. 2:**
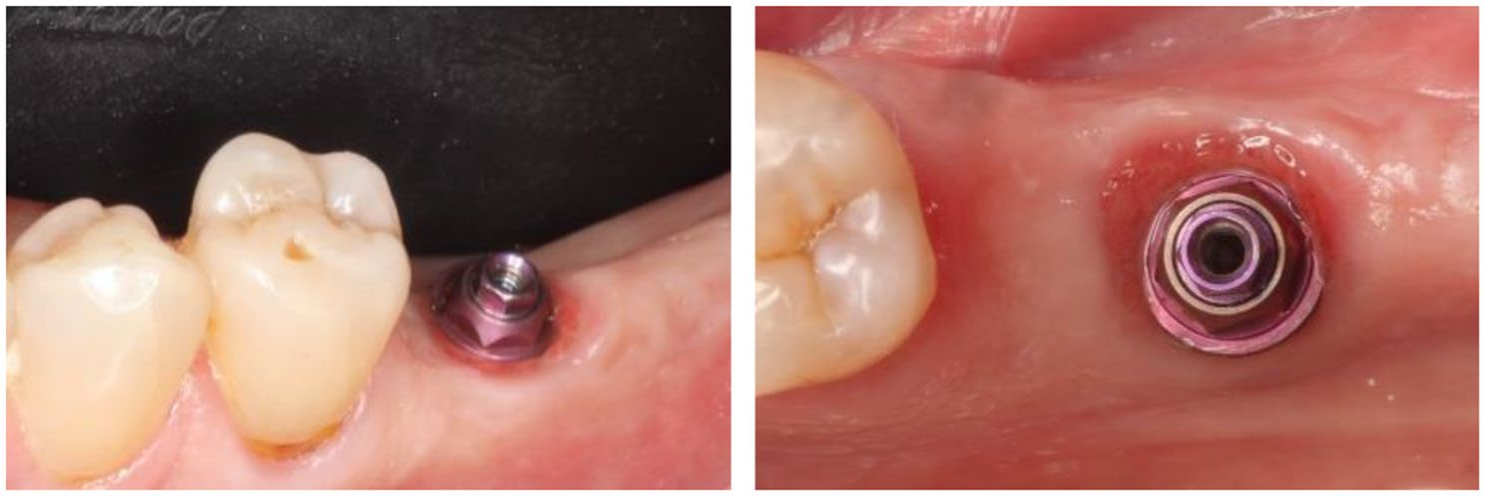
Views of the emergence profile after unscrewing the crown. **a**) Vestibulo-occlusal view of the soft tissue of the emergence profile after 3 months of function. **b**) Occlusal close-up view. The mesial gingiva participated mostly to the emergence profile conditioned under the crown

After 6 weeks of soft tissue healing (Fig. 1a) an intraoral scan (IOS) was taken with the healing cap in place (IOS#1). Immediately after, the healing cap was removed and an IOS was taken with the scan-body placed on top of the transepithelial abutment. The intra-oral scan was sent to the dental lab and the dental technician designed and produced by CAD-CAM a screw-retained crown (DentalCAD, Exocad, Darmstadt, Germany; MillBox CIMsystem, Balsamo, Italy). The single crown was affixed to the transepithelial abutment; strains exerted by the prosthesis on the healed gingiva led to various intensities of gingival blanching (Fig. 1b). After 30 min, the presence of blanching was checked as having completely vanished (Fig. 1c). Following prosthesis delivery, a second IOS (IOS #2) was taken with the crown in place. After 3 months of function and soft tissue conditioning, the crown was unscrewed and a third IOS (IOS#3) was taken; this recorded the emergence profile that was shaped under the implant-supported crown (Fig. 2a, b).

### The digital protocol

The digital merging procedure previously described by Szmukler-Moncler et al. [17] was applied. On top of the 3 above mentioned IOSs, it involves on one hand the STL (Standard Tessellation Language) files of the healing cap, the transepithelial abutment and the implant, all provided by the manufacturer and on the other hand the STL file of the crown designed by the dental technician.

**Fig. 3:**
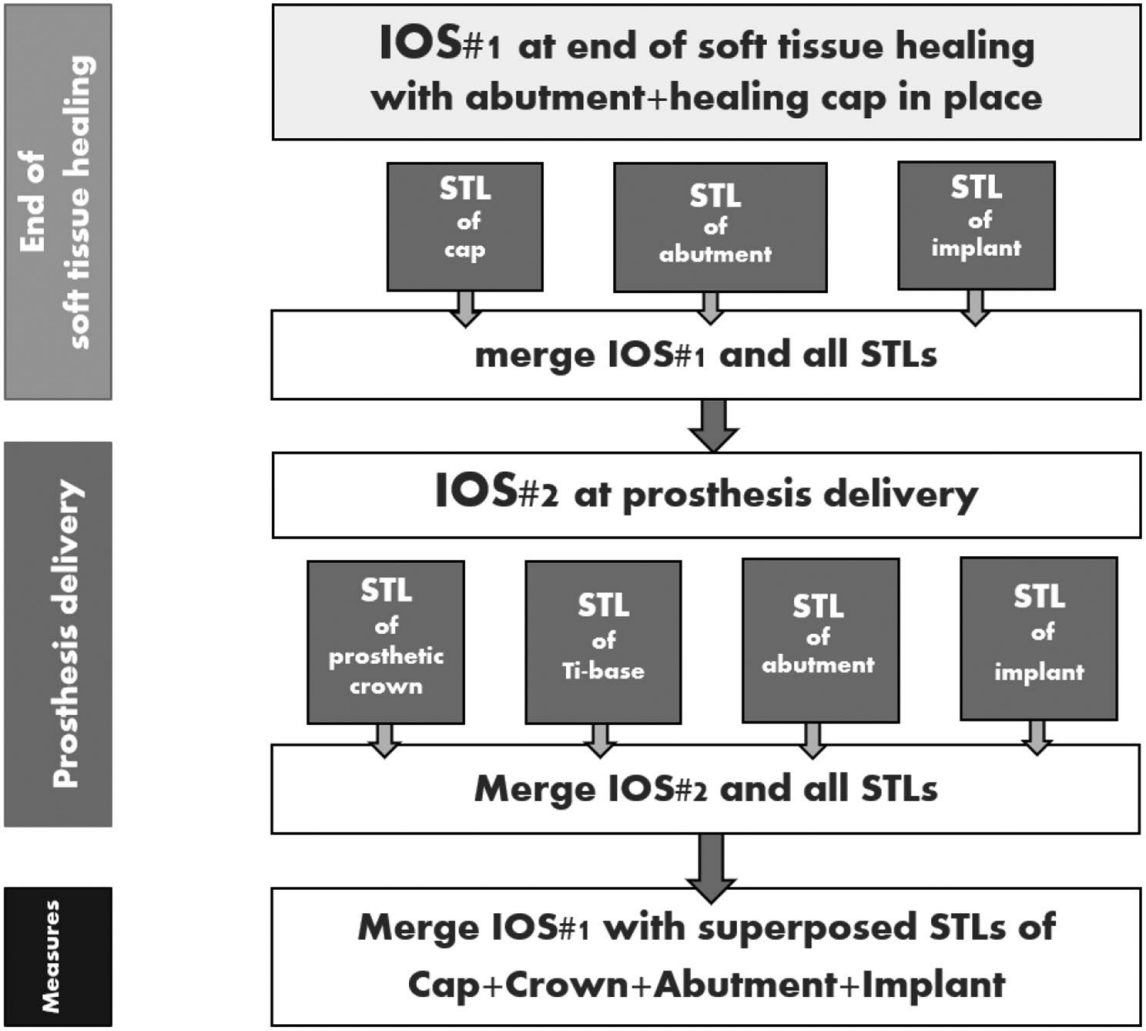
>Diagram showing the various superpositions and merging steps. IOS#1 was first handled with the various STLs; the aim was to merge appropriately the healing cap with IOS#1. Then, IOS#2 was handled with the various STLs; the aim was to merge the STL of the crown with IOS#2. This succession of superpositions and merging allows reading the distances the crown compresses and stretch the gingiva

**Fig. 4:**
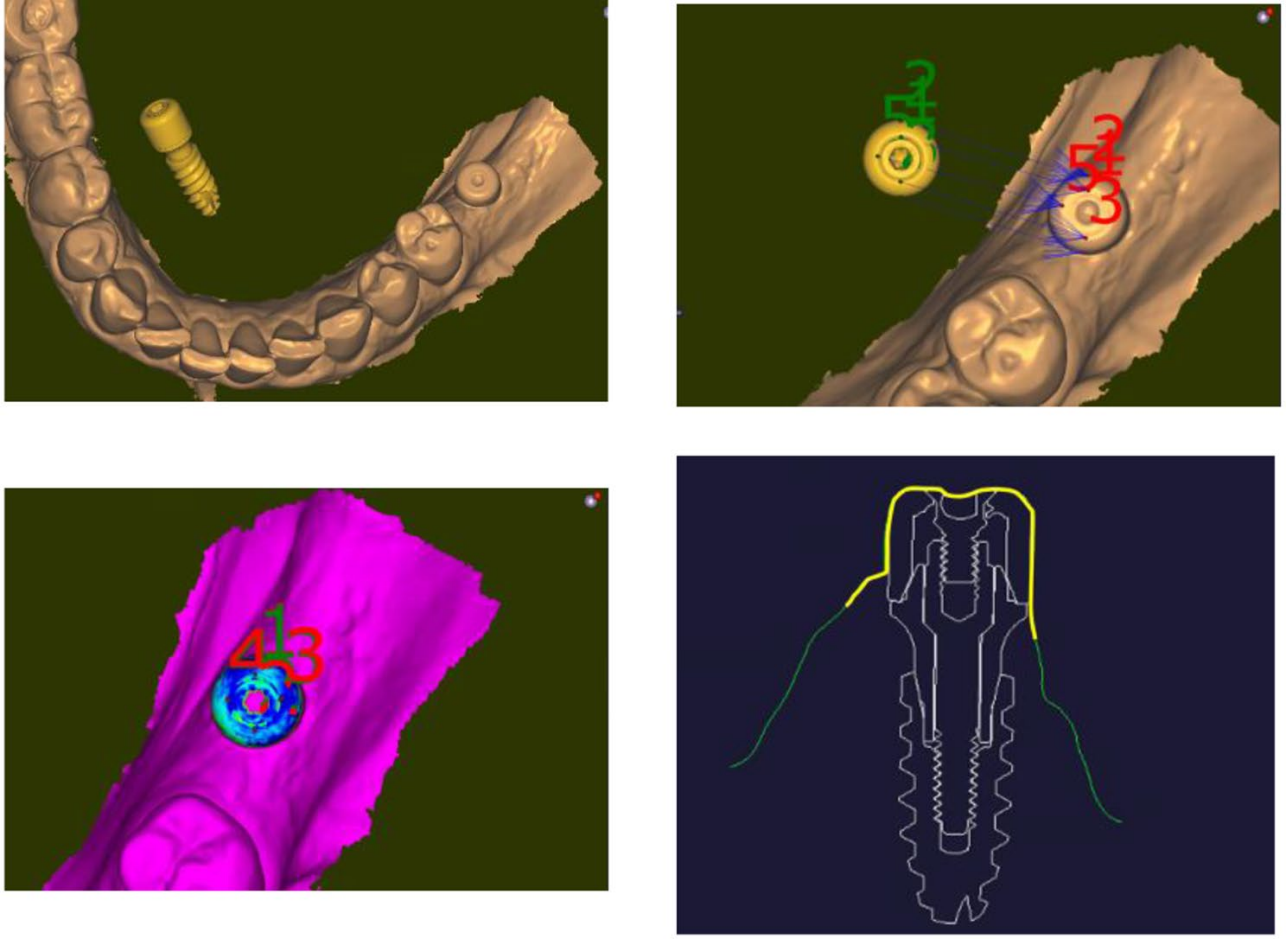
Merging the healing cap with IOS#1. **a**) Importation of the STL of the healing cap, the transepithelial abutment and the implant while preparing the merging with IOS#1. **b**) Steady point referencing in preparation of the automatic merging between the STL and IOS#1 by selecting 5 similar reference points. **c**) Successful result of the automatic merging of the STL of the healing cap. **d**) Vestibulo-lingual section of the merging of the STL of the healing cap-transepithelial abutment-implant with IOS#1. The green line refers to IOS#1; the yellow line highlights the external delimitation of the healing cap and the further gingiva. Note how the healing cap seats on the transepithelial abutment

Figure 3 lists the various steps of the digital protocol. First the STL of the healing cap, the transepithelial abutment and the implant were superposed according to the drawings of the manufacturer in a single STL (Fig. 4a); then, it was merged with IOS#1 until matching (Fig. 4a-c). Figure 4d represents a section of the merging of the superposed implant items with IOS#1; it shows how the healing cap seats on the transepithelial abutment.

**Fig. 5:**
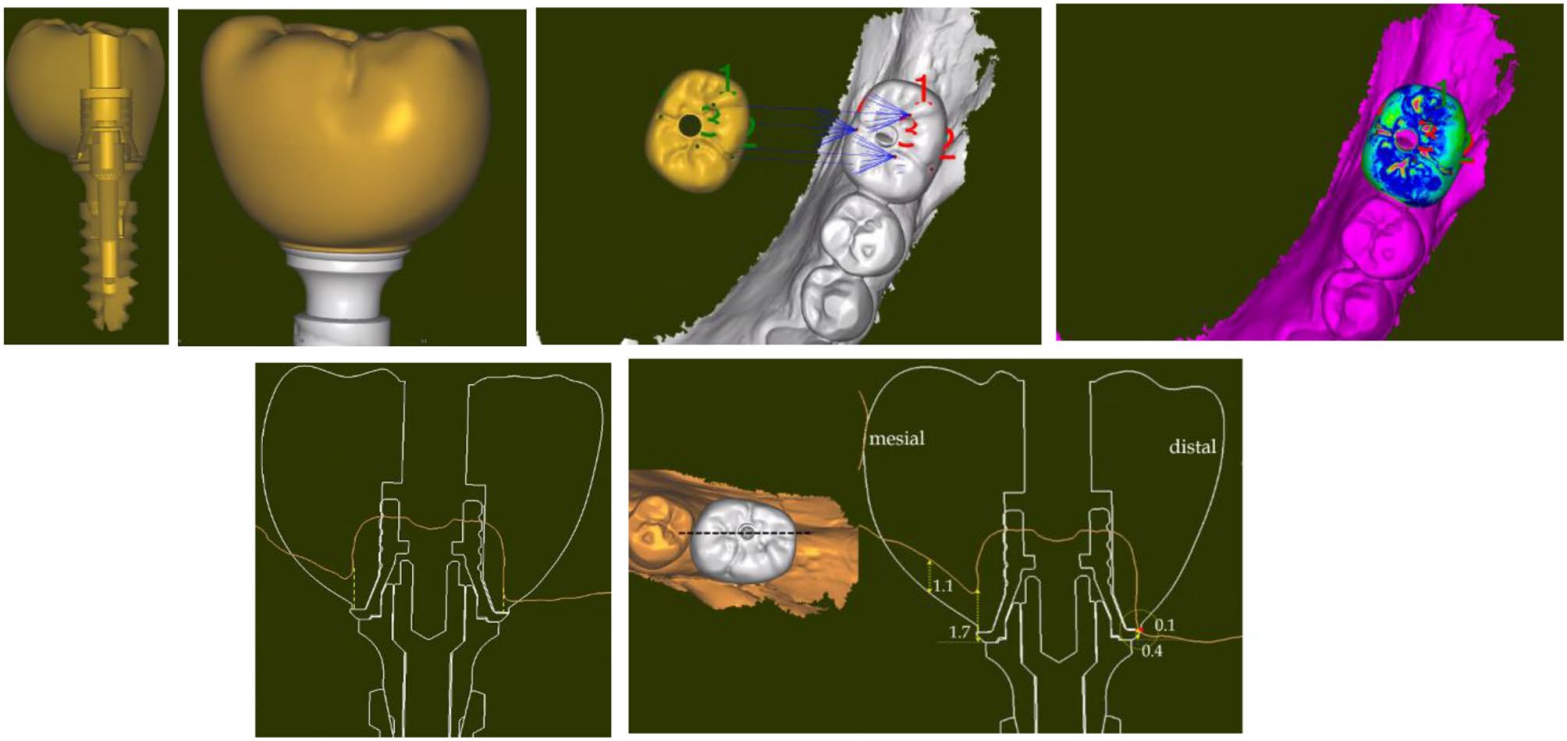
Merging of the crown with IOS#2. **a**) Image of the superposition of the crown, the Ti base, the transepithelial abutment and the Top DM implant. **b**) Close-up of the connection between the crown and the transepithelial abutment as read on the STL. **c**) Referencing the STL of the crown in preparation of the automatic merging with IOS#2. **d**) Successful result of the automatic merging of the STL of the crown. **e**) Mesio-distal view of the superposition of the STL of the crown and its related items with IOS#1 (in orange). The STL of the healing cap is not shown here in order to allow for a better reading of the figure. The yellow dot lines are delimiting the external envelope of the healing cap. An apical limit of the crown below the orange line means that the crown squashes and displaces the healed gingiva. The distance of the squeezing can be measured at every point of the gingiva. **e**) Measure of the displacement distances the crown brought to the healed gingiva. The mesio-distal axis where the displacements were measured is shown in the occlusal view of the crown (left). The highest displacement distance the crown is triggering on the gingiva is 1.7 mm on the mesial side. On the distal side, pressure of the crown was over 0.4 mm (in yellow) and stretching was over 0.1 mm (in red)

As for the prosthetic part, the first step was to superimpose the STL of the crown with the STL of the titanium base of the crown, the transepithelial abutment and the implant in a single STL (Fig. 5a, b); this STL was then merged with IOS#2 to place the crown and its related items in its correct position (Fig. 5c, d). Then, this STL was merged with IOS#1; it now allows measuring the distance the crown squashes and stretches the gingiva (Fig. 5e, f). Completing this procedure of successive merging and superpositions allows measuring the distance the crown is squeezing the peri-implant gingiva and stretching it at every place of the emergence profile, from the most coronal part of the crown to the most apical one.

The measurements on the digitally merged files were performed at the sites of the various blanching intensities (Fig. 1b), at the vestibulo-mesial side where the gingiva exhibited the most severe blanching, at the mid-vestibular side where blanching was moderate, at the vestibulo-distal side where blanching was absent, on the mesial side where blanching was severe and on the distal side where blanching was absent.

**Fig. 6:**
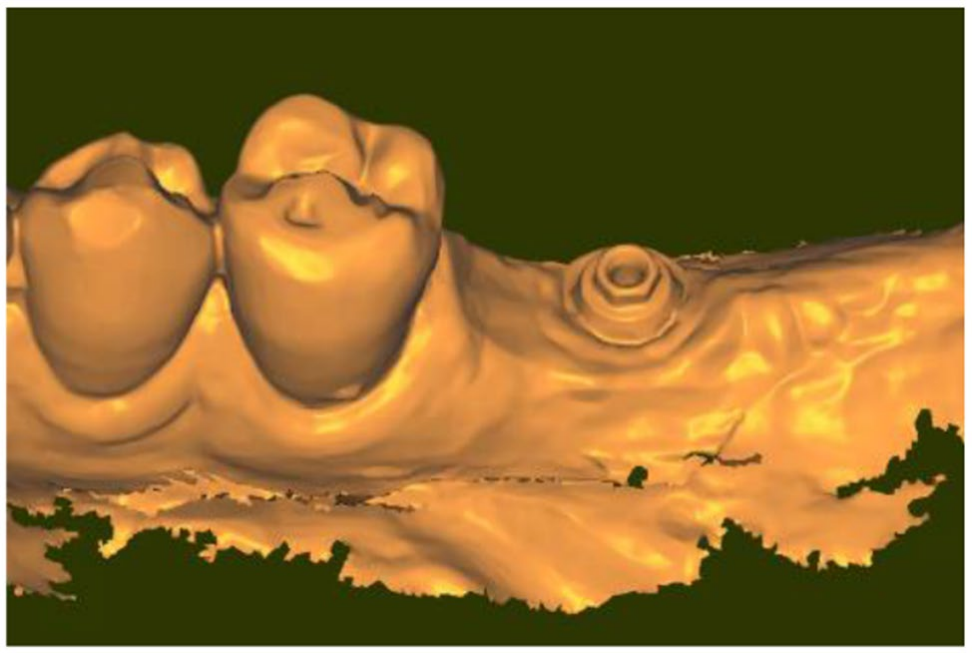
View of IOS#3 taken after unscrewing the crown. Note the hexagonal head of the transepithelial abutment and the emergence profile that has been conditioned under the crown

After 3 months of function and soft tissue conditioning of the emergence profile, the crown was unscrewed and a scan (IOS#3) was taken to get the exact shape of the emergence profile (Fig. 6).

To visualize the places where the gingiva was strained in order to lead to the actual emergence profile, IOS#1 and IOS#3 were superposed while subjecting IOS#3 to a partial transparency.

The IOSs were performed with the Trios 3 scanner (3Shape, Copenhague, Denmark); the superposition and merging steps as the distance measurements were performed with the Exocad software (Exocad, Darmstadt, Germany).

### Results of the measurements

**Fig. 7:**
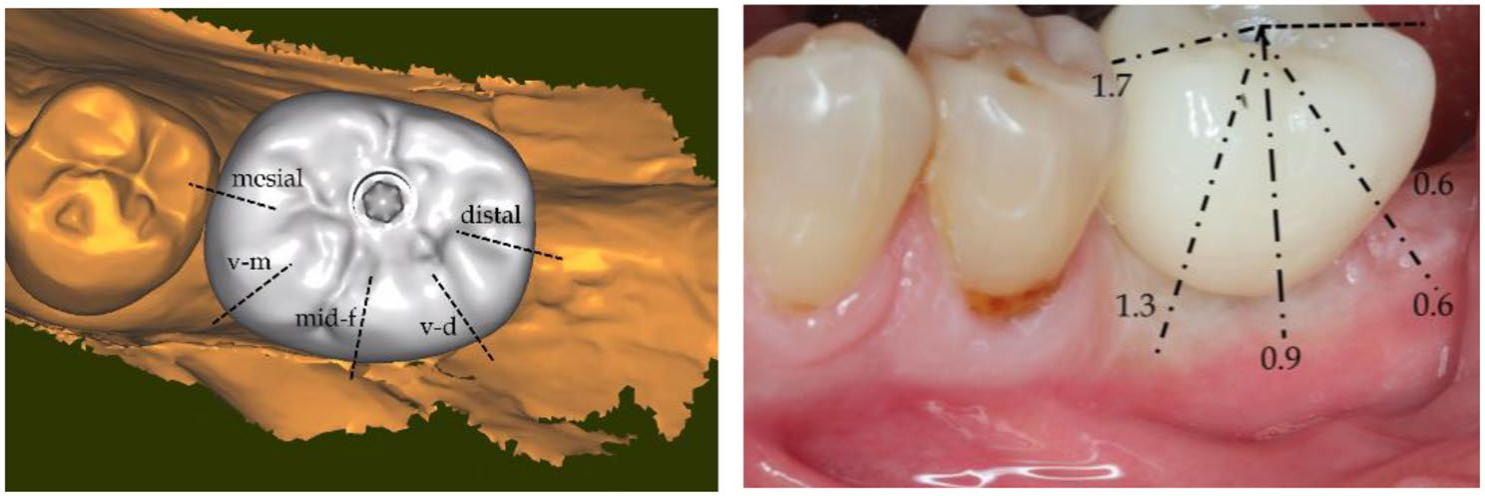
Results of the measurements. **a**) Occlusal representation on the crown of the plans where the displacement distances were recorded. Five sites have been measured; each one corresponds to a distinct blanching response of the gingiva to the strains yielded by the prosthetic crown. **b**) Measurements corresponding the various blanching intensities of the gingiva during crown delivery. On the vestibular site, the most intense bleaching was due to a displacement of 1.3 mm, the moderate blanching responded to a pressure of 0.9 mm. No blanching was observed when compression was 0.6 mm. On the mesial side, blanching is masked by the crown but the pressure was there the highest with 1.7 mm

The soft tissue displacements caused by the crown that led to the various intensities of blanching are shown in Fig. 7a and b. Severe blanching appeared when the crown strained the gingiva over a distance of 1.3 to 1.7 mm; moderate blanching was seen upon a displacement of 0.9 mm and lack of blanching was found up to a displacement of 0.6 mm.

**Fig. 8:**
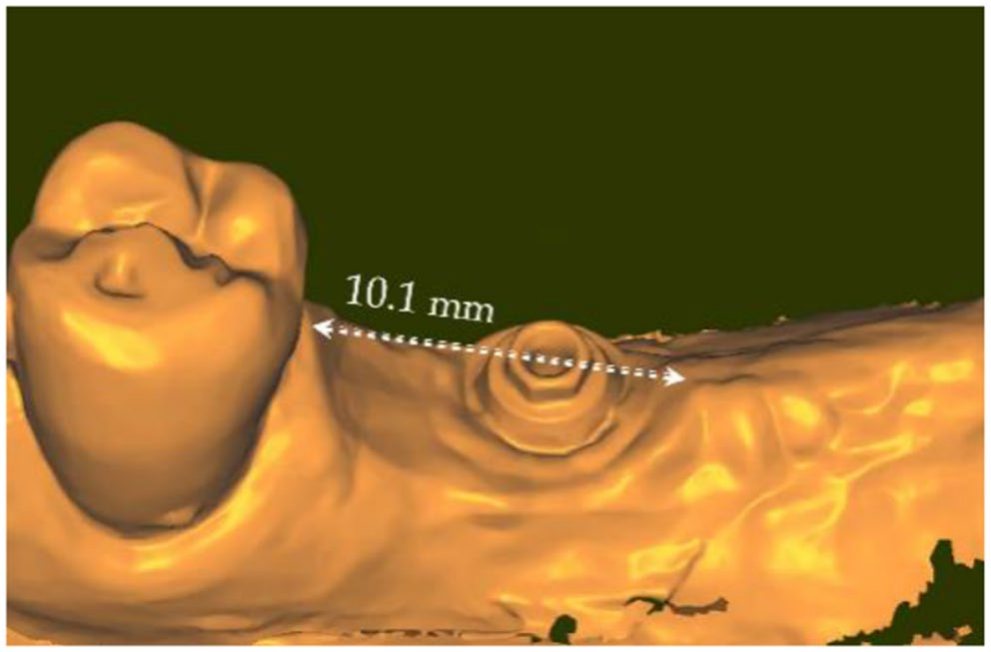
Mesio-distal measurement of the emergence profile read on IOS#3. The mesio-distal distance of the emergence profile is 10.1 mm. It has been enlarged compared to the initial Ø 5.0 mm emergence profile reached around the healing cap

**Fig. 9:**
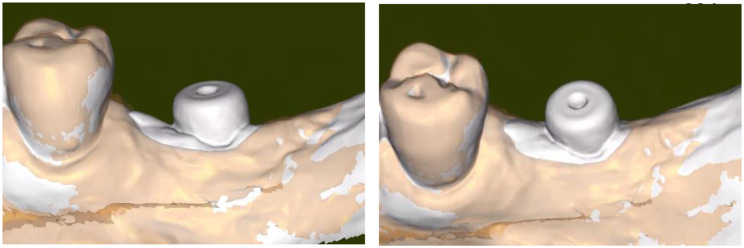
Superposition of IOS#1 and IOS#2 showing the topography of the soft tissue conditioning under the crown. **a**, **b**. Vestibular and vestibulo-occlusal view of the location of the strained gingiva. IOS#3 (in brown) was rendered partially transparent to allow identifying the initial topography of the gingiva (in white) given by IOS#1. These images reveal that pressure was mostly exerted by the crown on its mesial side and much less on its vestibular one

The mesio-distal length of the emergence profile was 10.1 mm (Fig. 8) and the vestibulo-lingual width was 5.9 mm; this is to compare with the Ø 5.0 mm external diameter of the healing cap of the transepithelial abutment. This means that the initial emergence profile shaped by the healing cap was increased by twice in the mesio-distal direction. Figure 9a and b are showing the evolution of the topography of the gingiva from the end of the healing period to its final conditioning under the crown. They are evidencing that, in the present case, the gingiva (in white) was mostly squeezed in the mesial portion of the actual emergence profile.

## Discussion

The success of implant therapy is now judged on long-term esthetic results; this requires maintaining the health of the supra-crestal soft tissues over time. Every step that participates in optimizing the gingival response must be acknowledged and nurtured. The surgical steps of thickening the gingiva have been extensively addressed [18]; however, much less attention has been paid to the prosthetic step in which, after removing the circular healing abutment, the temporary or definitive prosthetic crown is attached to the implant neck while assaulting the delicate gingival mucosa in all directions, through squeezing and stretching [5–8, 10–14]. In fact, as far we are aware, there is no literature at all about how much strain exerted by the prosthetic crown is leading or not to gingiva blanching.

To avoid insulting the soft tissue architecture in the esthetic anterior area when transiting from a narrow circular emergence profile to the large one of the central incisor crowns, soft tissue conditioning has been advocated by a gradual enlargement of the emergence profile with successively larger circular healing abutments [5]. To deal with this issue, Wittneben et al. [5] advocated a dynamic compression procedure in the anterior maxilla; it consists into delivering an overcountoured provisional crown that will be gradually reshaped by adding and removing material at the proximal and central sides. They mentioned that blanching while delivering the temporary crown should not extend by more than half of the neighboring teeth and be present for no more than 20 min [5]. However, this empirical statement does not provide details of how much the crown is allowed to strain the delicate circular emergence profile installed by the rounded healing abutment. This information is critical to the lab technician to help him/her designing appropriate prosthetic crowns.

The present digital protocol allows for a straightforward measurement of the displacement distances that translate into the strains that are exerted at every spot of the emergence profile of the implant-supported crown. With this tool at hand, it becomes possible to study the correlation between blanching and compression/stretching and then define thresholds of acceptable strains. This correlation is worth to investigate because it can be assumed that placement of an implant crown without prior emergence profile modulation might increase the esthetic and functional risks for inadequate mucosa architecture due to uncontrolled pressure application [6].

Above all, such data should be significant to the dental technicians; they will know how much it is possible for them to strain the gingiva with the aim of conditioning it to the shape of the crown they are designing. Until now, this is done based on a too vague assumption of the emergence profile [6]. The dental lab would then require receiving from the prosthodontist an IOS with the healing abutment in place and design the crown accordingly on the dedicated software.

The data of the present case showed that a pressure of the crown of up to 0.6 mm did not lead to blanching while a pressure of 1.3 mm and above led to a severe blanching; moderate blanching was found under a 0.9 mm pressure. The crown created an emergence profile of 10.1 mm in the mesio-distal axis that is more than the double of the Ø 5.0 mm healing cap, i.e. an enlargement of more than 5 mm. The gingiva was extensively pressured and stretched to reach this much larger emergence profile, especially on the mesial and vestibular sides (Fig. 9a, b).

The strains leading to blanching are assumed to highly depend on the horizontal and vertical dimensions of the gingiva, its stiffness and the biotype; therefore, no numerical conclusion of how much displacement of the healed gingiva leads to blanching can be drawn from this unique case. Future studies correlating a large number of cases involving blanching and the related strains will enable the scientific community to better address this issue; the use of spectrophotometry [19] or any other available method to assess objectively the color of the gingiva [20] will be determinant. Also, one could investigate any relationship between soft tissue recession and initial blanching of the gingiva. Finally, histologic studies should be welcome to better understand the cellular behavior of the supraimplant soft tissues during compression and its release [6].

## Conclusion

In conclusion, aim of this paper was to share a digital method that combines the superposition and merging of IOSs and STLs of the various hardware elements involved in implant therapy. It allows measuring the displacement distances the prosthetic crown enforces on the healed peri-implant soft tissues that translate into strains and that lead or not to gingival blanching, at every spot of the marginal gingiva.

The data issued from this study case were that severe blanching was observed when a displacement of 1.3 mm and above was yielded on the gingiva by the prosthesis; a pressuring distance of 0.9 mm led to moderate blanching. No blanching was observed up to a displacement of 0.6 mm. From this single case no numerical conclusion can be drawn; but with this digital tool at hand, large cohort studies are warranted to pour some light on this poorly addressed issue.

## Data Availability

No datasets were generated or analysed during the current study.
